# Water uptake mechanism and germination of *Erythrina velutina* seeds treated with atmospheric plasma

**DOI:** 10.1038/srep33722

**Published:** 2016-09-27

**Authors:** Clodomiro Alves Junior, Jussier de Oliveira Vitoriano, Dinnara Layza Souza da Silva, Mikelly de Lima Farias, Nadjamara Bandeira de Lima Dantas

**Affiliations:** 1 Plasma Laboratory Applied to Agriculture, Health and Environment (LabPlasma), Federal Rural University of the Semi-arid Region, Mossoró, RN, Brazil; 2 Postgraduate Program in Natural Resources, University of Rio Grande do Norte State, Mossoró, RN, Brazil.

## Abstract

The effect of plasma applied to mulungu (*Erythrina velutina*) seeds was studied to verify its influence on the germination, water absorption, wettability and structure of the seeds. The plasma jet used in this study was produced by dielectric barrier discharge (DBD) in a helium gas flow of 0.03 L/s at a distance of 13 mm for 60 s. The plasma treatment significantly affected the seed germination rate, which was approximately 5% higher than that of the untreated group. Micropyle and hilum contributed a greater proportion to uptake. When sealed in the hilar or micropyle regions the amount of water absorbed into the seed decreased approximately 75% compared to the unsealed seed. This difference suggests that these two regions together act cooperatively in the water absorption. However, when plasma treated seed was blocked in the micropyle region, water absorption was higher higher than in seeds blocked hilum. This difference suggests that the plasma treatment changed the wettability of the hilum more effectively than it changed the micropyle. These results indicate that plasma can significantly change the hydrophilicity, water absorption and percentage of seed germination in *E. velutina*.

*Erythrina velutina* (mulungu) is a xerophilic plant that is native exclusively to a Brazilian biome called Caatinga, located in the semiarid region of Brazil. This plant is commonly used for medicinal purposes, lumber, handicrafts, ornamental use, and now as a component of agroforestry systems^1^. Xerophilic seeds such as *E. velutina* exhibit dormancy (resistance to germination) as a mechanism of survival and adaptation to challenging climatic conditions^2^. Physical dormancy is attributable to the impermeability of the seed coat, which comprises cells rich in hydrophobic substances (cutin, lignin, quinones, suberin and wax) protecting the embryo. Physical or chemical treatments to overcome tegumentary dormancy include water immersion, chemical and mechanical scarification, and growth regulators. Among these treatments, mechanical scarification with sandpaper has been the most efficient in breaking dormancy and promoting *E. velutina* seed germination^3^.

However, mechanical scarification presents problems such as reduced vigor, increased rates of microorganism infections, and an increased probability of abnormality in seedlings, shoot development and biomass production^4^,^5^,^6^.

Plasma produced by dielectric barrier discharge (DBD) is a fast, economical and pollution-free alternative method^7^ that has shown positive results for breaking dormancy^8^,^9^ and inactivating microorganisms^10^,^11^. Nevertheless, very few studies have investigated the mechanism underlying the modification of the wettability and water uptake into seeds; furthermore, few have identified the materials involved or have planned experimental strategies to modify the surface using plasma. In this study, *E. velutina* seeds were investigated to identify the mechanisms involved in the water absorption pathway and to elucidate the conditions necessary for the plasma DBD process.

## Results and Discussion

### Germination test

Plasma treatment affected the seed germination rate and significantly affected the germination potential, index and emergence speed index (Table 1).

The maximum germination rate (GR) was obtained for the plasma-treated seeds; this difference was small but significant when compared with the untreated seeds. It was apparent that the plasma-treated seeds germinated more uniformly. The germination potential (GP), which is the total number of germinated seeds in the first 5 days of sowing, was the same for treated and untreated seeds, but the standard deviation for the treated seeds was smaller (i.e., they were more uniform). Both the sum of germinated seeds per respective day (IG) and the sum of the ratios of emerged plants by the number of days (ESI) were higher when treated with plasma. This result indicates that the seeds treated with plasma germinated and emerged primarily in the first days after sowing.

**Figure 1:**
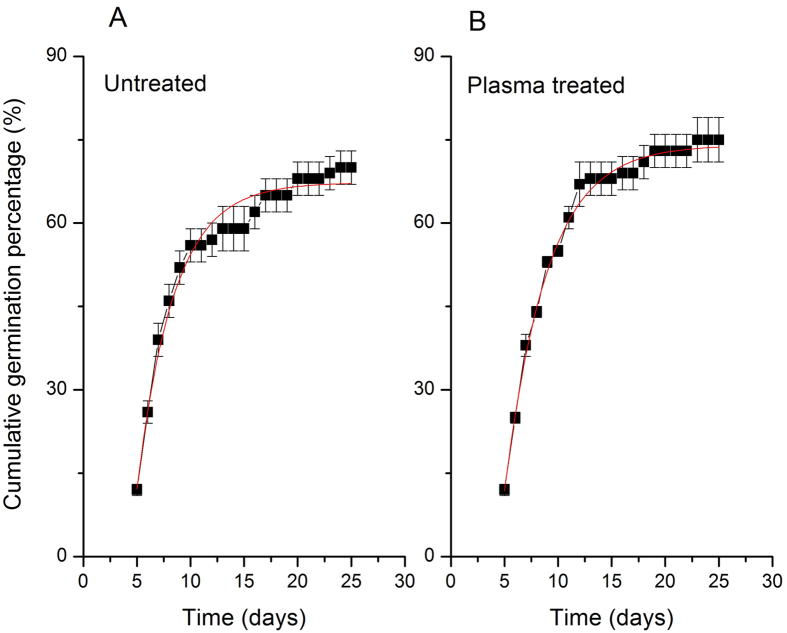
Germination curves calculated using the Richard’s fitting funcion. (**A**) seeds untreated, (**B**) seeds treated.

The changes induced by plasma germination kinetics can also be represented by Richards curves (Fig. 1A,B). Taking into account the setting parameters between Richards and sigmoidal curve was obtained population parameters V_i_, M_e_, Q_u_ and S_k_ (Table 2).

As expected, the viability values (Table 2) and germination rate (Table 1) were similar (Table 1), since this discloses the maximum percentage of germinated seeds. The median values (I) of the treated seeds in plasma were similar to untreated seeds, indicating that the process did not alter the biological character of espécie^12^. It is also found that these treated seeds, despite the germination process to be later (higher value S_k_) have less dispersion in germination time values (lowest value Q_u_).

Thus, the plasma treatment had a positive effect on the germination of *E. velutina*. Studies of different plant species, for example, in the seeds of soybean^7^, beans^13^ and poppy^9^, found that plasma treatment significantly increased seed germination.

Because of the action of the plasma on the structural characteristics of seeds, it is probable that when seed coat formation is potentially hydrophobic, the altered seed coat can increase the hydrophilic ability of the seed and eventually improve the water uptake. Seeds immersed in plasma are subjected to attack by oxygen radicals and are bombarded by ions, resulting in seed coat erosion and etched/eroded surfaces.

The wettability of a seed can be reflected by the apparent contact angle value. Plasma treatment can change the chemical structure and roughness of the surface, leading to a dramatic change in the wettability behavior of the seed. Water uptake is accompanied by an increase in the absorption of nutrients responsible for promoting the growth of seedlings, as noted in a previous study^7^ when soybean seeds were treated with cold plasma.

### Seed apparent contact angle

**Figure 2:**
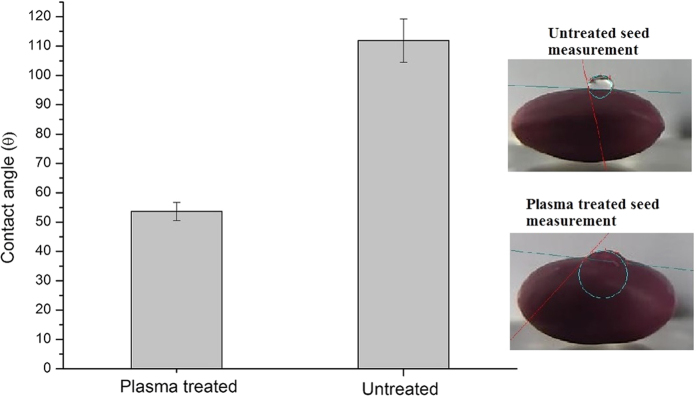
Wettability test in untreated and plasma-treated *Erythrina velutina* seeds.

The present study, similar to a previous study of radiofrequency cold air plasma treatment^14^, found that cold plasma treatment significantly decreased the apparent contact angle (Fig. 2).

The apparent contact angle of the seeds untreated with plasma was 112°. Compared with the untreated seeds, those treated with plasma exhibited decreased apparent contact angle by 48% (p < 0.05). The lowest apparent contact angle occurred when using the plasma treatment.

There are still many unresolved issues with respect to both the plasma action mechanisms in seeds and the water soaking mechanisms. The latter is certainly associated with the structure and composition of the seed. A study of the *E. velutina* seed structure was conducted to identify the important components of water absorption.

### Structure of the seed

**Figure 3:**
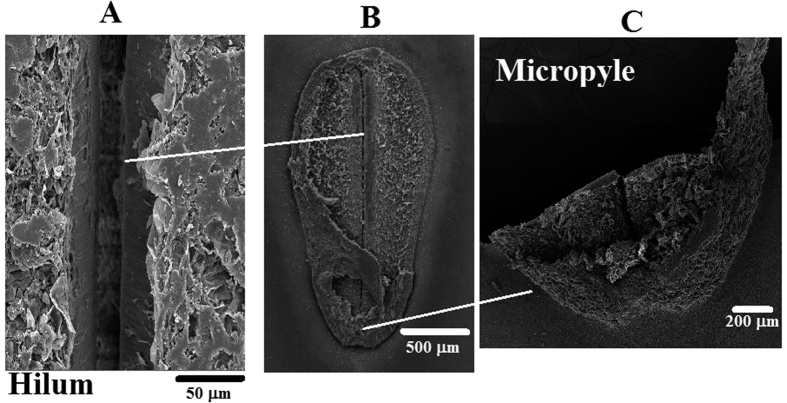
Picture of the *E. velutina* seed, showing details of the parts that were examined.

It is common for species belonging to the family Fabaceae, such as *E. velutina*, to have a testa formed of impermeable cells. The testa is generally arranged as a palisade with thick and lignified secondary walls, whereas the macrosclereids, the most common cells, are filled with hydrophobic substances such as cutin, quinones, insoluble pectins, suberins and waxes^15^. The hilar region of *E. velutina* (Fig. 3B) comprises shows details the micropyle (Fig. 3C) and hilum (Fig. 3A). The micropyle has thick endothelial cells that can expand and increase the absorption of water.

The hilum comprises a layer of palisade cells bounded externally by remaining subcuticular tissue (Fig. 3A) as well as an extra-hilar layer adjacent to the palisade layer (a transverse subcuticular layer) and a more external cuticular layer^15^. Structural analysis of the hilar region of *E. velutina* (Fig. 3) revealed that treated seeds of this region remained intact in all tissues.

However, when treated with plasma, the micropyle and hilum of the seeds changed; the hilum increased the amount of water absorbed, and the micropyle exhibited a more open configuration.

The influence of water absorption in different anatomical regions was studied. The results showed that both the micropyle and hilum were the main regions involved in the absorption of water.

However, *E. velutina* seeds showed physical dormancy; the testa had hydrophobic substances in the outer layer that decreased the absorption of water. The first step in the process consisted of absorbing water by forcing open the endothelial cells of the micropyle, allowing water into the inside of the seed. Seeds that were soaked for 48 hours in bromophenol blue showed the blue color only in the region of the micropyle, indicating that this region was the first to absorb water.

### Water absorption: preferential region and pathway

This experiment evaluated the uptake of water when seed parts are sealed by glue. It is clearly showed pathway and preferential water absorption regions of *E. velutina* seeds, When analyzing the individual contributions of the different regions of the seed surface (Tables 3 and 4).

For untreated seeds (Table 3), a significant (p < 0.05) reduction in water absorption was observed with application of silicone glue to the hilar region versus that in seeds without glue. The seeds without glue absorbed 18.09% of their weight, whereas the seeds sealed with glue on the hilum absorbed only 5.14%. This difference indicated that the hilum has an important contribution to water absorption. For the micropyle covered with glue, we verified that the absorption was only 4,06%, meaning also the important of this region in the absorption of water, as already observed in the literature^15^. Thus, micropyle and hilum contributed a higher proportion to the imbibition.

When the plasma treatment was applied to seeds, a reversal of the absorption capacity between the hilum and micropyle occurred, with the latter providing a lower contribution to absorption (Table 4). This finding indicates that the plasma treatment was more effective in the hilum than in the micropyle with respect to wettability.

In general, the period between soaking and germination ranges from 6 to 21 days, and the seeds can respond to a germination stimulus during this period if the signal through the roots is not received during imbibition^16^. Therefore, the accumulation of large amounts of water in the mucilaginous endothelium is considered an adaptive advantage of species to dry environments.

The interaction of cells with plasma may enhance the enzyme activity and accelerate the decomposition of internal seed nutrients, which may contribute to the increased use of the reserve of seeds as well as the growth of seedlings. A previous study^17^,^18^ confirmed this finding by observing that the plasma promotes increased activity of amylase and protease in the metabolism of soluble sugars and protein, which is essential to the germination process.

## Materials and Methods

**Figure 4:**
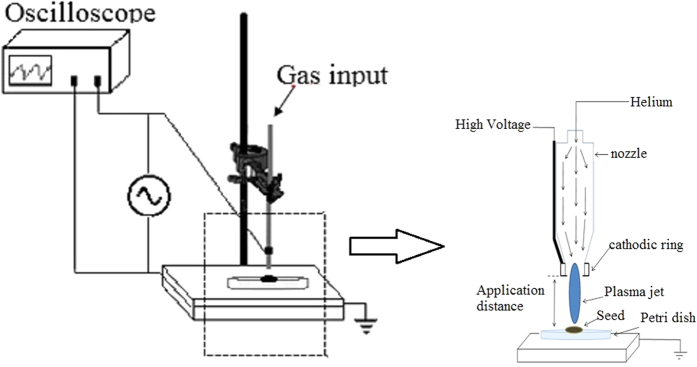
Schematic of the apparatus used for plasma generation.

Seeds of mulungu (*E. velutina*) were obtained in 2013 from Lagoa Grande/PE-Brazil. The research was carried out at the Federal Rural University of the Semi-arid Region, Mossoró-RN, Brazil (5° 11′ 31″ S 37° 20′ 40″ W, altitude 16 m). Figure 4 illustrates the experimental apparatus used to treat the seeds using DBD plasma.

Plasma jet produced by helium gas discharge in a flux of 0.03 L/s was applied to each seed at a distance of 13 mm for 60 s. The following conditions were applied: voltage of 10 kV, frequency of 750 Hz and power of 150 W. Plasma diagnostic by Optical Emission Spectroscopy – OES has been utilized and species such as N2+ (391,4 nm), O (777,4 nm) and O (842,6 nm) have been monitored during plasma treatment. During application of the plasma jet on individual seeds, it was made small rotational movements to ensure greater uniformity that was confirmed by low dispersion of contact angle value.

### Water absorption: preferential region and pathway

**Figure 5:**
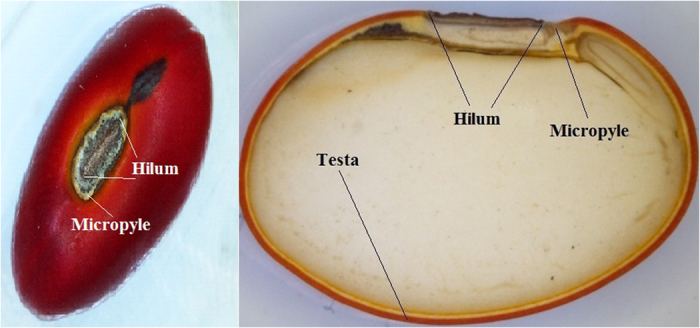
Picture of profile and cross section of *E. velutina* seed showing the parts that were sealed in the soak test. The testa corresponds to the remaining surface seed (red wrap).

The primary purpose of this study was to characterize both the preferential region of water uptake and the water pathway. For this purpose, specific parts were sealed with glue. Six groups of seeds were used to determine the preferential region of water uptake—three untreated and three treated with plasma—with each group containing 20 seeds (4 × 5). The seeds group untreated were divided into seeds without glue and seeds with glue applied to the hilum or micropyle. For the treated group, the following seed parts were isolated (Fig. 5): hilum, micropyle and hilum + micropyle. The water uptake was calculated by weighing before and after each seed absorption test.

To identify the path followed by the water, the seed was sectioned longitudinally and transversely and was observed using a stereoscopic microscope. The outer part of the seed was coated with cotton soaked in water. The penetration of water into the seed surface was then recorded.

### Wettability test

Using the sessile drop method, the contact angle was measured. Thirty seconds after adding one drop of distilled water to the seed surface, the image was captured using a model VP 540s camera (Intelbrás). The contact angle was determined according to the average of the seven seeds using SURFTENS software OEG GmbH (Germany)^19^. The values of contact angles formed by water with the cutaneous surface of the seed were submitted to descriptive statistics (arithmetic mean and standard deviation).

### Germination test

The germination test used 100 seeds treated with plasma and 100 untreated seeds divided into four replicates of 25 seeds. These were seeded in aluminum trays containing washed and sterilized sand and received daily irrigation to keep the substrate at 70% of field capacity. The first count was performed after the 5th day of seeding^20^.

The experiment was planned with a completely randomized design with four replications. The following variables were evaluated: germination rate (GR), germination potential (GP), germination index (Gi) and seedling emergence speed index (ESI).

- GR (%) = (number of seeds germinated in 25 days/number of seeds) *100%;

- GP (%) = (number of germinated seeds in 5 days/number of seeds) *100%;

- Gi = ∑ (number of germinated seeds on the day/germination days);

- ESI = (E1/N1) + (O2/N2) + … + (En/Nn)^21^.

where,

E = number of normal seedlings counted in scores;

N = number of days from sowing to the 1st, 2nd … 25th evaluation.

The experimental data of the cumulative percentage of germination was plotted versus time. Fitting of experimental data by Richads’ Equation^22^ and comparing with the sigmoidal or logistical curve, modeling population growth, supplied the best values of the fitting parameters, in Which M_e_ (median) denotes the time of 50% germination and characterizes the rate of this process. The quartile deviation of germination Q_u_ Describes the team deviation range of the Richards ‘curve relative to M_e_, and S_k_ (skewness) represents the asymmetry of the Richards’ curve relative to the point inflection (mode)^12^.

### Statistical analysis

The statistical analyses for all methods had an entirely randomized design in a factorial arrangement. For the test that involved only soaking, the data were arranged in a factorial scheme (5 × 2): five seed regions with glue under two conditions (with and without plasma) with four replications. In other cases, analysis of variance as well as comparison of means using Tukey’s test (p < 0.05) by Sisvar^®^ software^23^ were performed, and results are presented as the mean with standard error (SE).

## Additional Information

**How to cite this article**: Alves Junior, C. *et al*. Water uptake mechanism and germination of *Erythrina velutina* seeds treated with atmospheric plasma. *Sci. Rep*. **6**, 33722; doi: 10.1038/srep33722 (2016).

## Figures and Tables

**Table 1:** Effects of plasma on the germination of *E. velutina*.

Treatment	Germination rate (%)	Germination potential (%)	Germination index	Emergence speed index
Untreated	70 ± 3.1^b^	12 ± 1.4^a^	9.42 ± 0.73^a^	2.97 ± 0.82^a^
Plasma treated	75 ± 3.8^a^	12 ± 0.8^a^	9.96 ± 0.69^a^	3.15 ± 0.88^a^

*Same letters in the same treatments indicate significance (p < 0.05) according to Tukey’s test.

**Table 2:** The population parameters Vi (viability), Me (median germination time), Qu (dispersion), and Sk (skewness) of the Richards equation for the germination of E. velutina.

	V_i_ (%)	M_e_ (days)	Q_u_ (days)	S_k_ (%)
Untreated	70 ± 3^a^	6.44 ± 0.63^a^	2.81 ± 0.18^a^	0.22 ± 0.02^b^
Plasma treated	75 ± 4^b^	6.79 ± 0.64^a^	1.81 ± 0.14^b^	0.43 ± 0.02^a^
*P* value	0.04*	0.12	0.002**	0.001**

^*^Difference at 5% level according to Tukey’s test;

^**^Difference at 1% level according to Tukey’s test.

**Table 3:** Preferential region and pathway in seeds of untreated *E. velutina*.

Untreated seeds			
	Wi (g)	Wf (g)	%
Seeds with glue in hilum	2.5550 ± 0.14	2.6863 ± 0.20	5.14^a^
Seeds with glue in micropyle	2.7100 ± 0.16	2.8200 ± 0.15	4.06^a^
Seeds without glue	2.3075 ± 0.12	2.7249 ± 0.58	18.09^b^

*Wi (initial mass); Wf (final mass). **Same letters in columns indicate similarity according to Tukey’s test (p < 0.05).

**Table 4:** Preferential region and pathway in seeds of treated *E. velutina*.

**Treated seeds**			
	Wi (g)	Wf (g)	%
Seeds with glue in hilum	2.7150 ± 0.21	2.8319 ± 0.30	4.31^b^
Seeds with glue in micropyle	2.4575 ± 0.09	2.6450 ± 0.38	7.63^a^
Seeds with glue in hilum and micropyle	2.6875 ± 0.10	2.6975 ± 0.10	0.37^c^

*Wi (initial mass); Wf (final mass). **Same letters in columns indicate similarity according to Tukey’s test (p < 0.05).
